# Supplementary figures and images for: Land suitability analysis in monocentric post-socialist city: Case of Ulaanbaatar, Mongolia
Source: PLoS One. 2024 Aug 15;19(8):e0308762. doi: 10.1371/journal.pone.0308762 (PMC11326617; doi:10.1371/journal.pone.0308762)

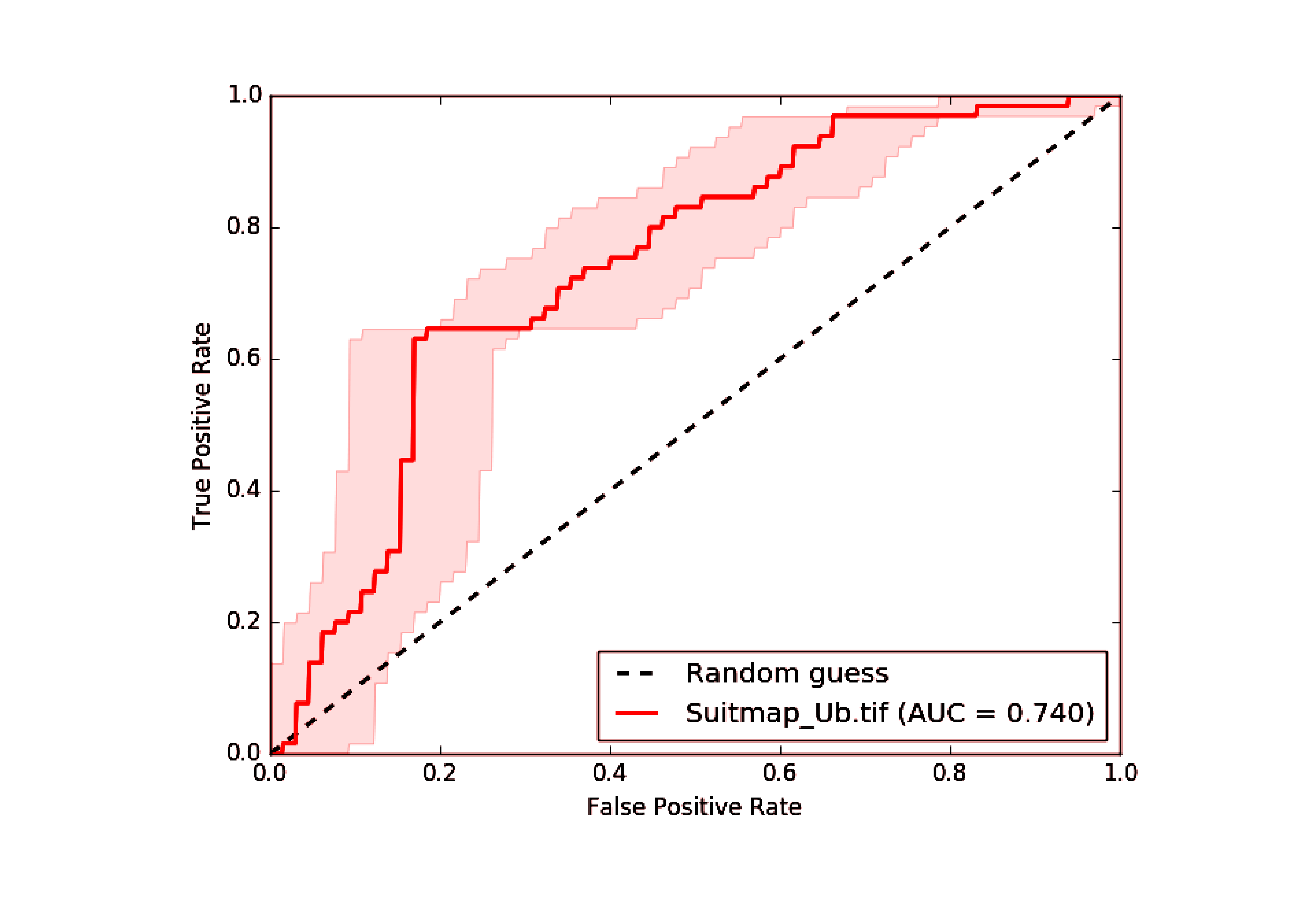

Supplement: S1 Fig — AUC is computed with the ArcSDM tool of ArcGIS software (version 10.6) from ESRI (http://www.arcgis.com). (TIF) [file pone.0308762.s001.tif]
