# Supplementary material for: Land suitability analysis in monocentric post-socialist city: Case of Ulaanbaatar, Mongolia
Source: PLoS One. 2024 Aug 15;19(8):e0308762. doi: 10.1371/journal.pone.0308762 (PMC11326617; doi:10.1371/journal.pone.0308762)
Supplement: Attachment — Submitted filename: Response to Reviewers.docx [file pone.0308762.s003.docx]

Dear Editor and Reviewers,

Thank you very much for the opportunity to submit our revised manuscript titled “Land suitability analysis in monocentric post-socialist city: case of Ulaanbaatar, Mongolia” co-authored with Galmandakh Boldbaatar, Gantulga Gombodorj, Dorligjav Donorov, Robert Andriambololonaharisoamalala, Myagmarjav Indra, and Myagmartseren Purevtseren to PLOS ONE.

We are extremely grateful to the two anonymous reviewers for providing detailed, thoughtful, and helpful comments, which led us to reconsider the explanations of the analysis, refocus the literature review and strengthen the paper. We believe that we were able to address almost all comments made by the reviewers. In our reply to reviewers, we explain how we addressed each comment.

We have addressed all the comments that are now reflected in our revised manuscript and inserted line numbers and highlighted significant changes by using GREEN text highlight color to expedite the checking process.

Here is a point-by-point response to the reviewers’ comments and concerns.

**Response for Reviewer #1**

**We appreciate the reviewers’ comments, which we find to be constructive and delivered professionally and appropriately. Thank you very much for your comments, each of which is addressed in the following. Our revised manuscript reflects your comments and suggestions. We have addressed all the comments that are now reflected in our revised manuscript and inserted line numbers and highlighted significant changes by using GREEN text highlight color to expedite the checking process.**

**Comments:**

*1- Identify 3-5 keywords under the paragraph of the abstract*

**Response:**	Thank you for your detailed and well-thought-out review. Our detailed responses to your criticisms and suggestions are provided below.

**Action: Keywords added in Line 44-45 as below:**

**Keywords:** urban multicriteria analysis; suitability evaluation; fuzzy logic; urban development; sustainable city

**Comments:**

*2- In the introduction part, the citations in years 2018-2020 are quite too old. Please find the more updated papers to support the paragraph of background of the study and problem statement.*

**Response:** We thank you for this valuable comment. Following your kind advice, the revised manuscript engages more strongly with existing findings published in interdisciplinary journals in recent years. Our introduction to the existing literatures now consists of more recent literatures.

**Action: New citations added Line 106, 115, 139 as below:**

Therefore, it is now widely admitted that analyzing and identifying the suitability of residential areas is very important for appropriate land use policies to improve the living condition of citizens for future urban development [9, 10, 15].

The process of land suitability analysis involved multicriteria analysis (MCA) and geographic information system (GIS), and the land suitability evaluation methods have significantly improved due to the development of data science and GIS. Multicriteria analysis manages complex factors for suitability analyses and has been used in various industries [16,17,18,19].

To minimize the subjectivity and mathematical shortcomings, the fuzzy logic theory, which was initially developed by Zadeh [34], was highly valued in the latest studies [24,35,36].

**New citations added on Reference in line 600, 608-614, 660-667 as below:**

# References

15.	Kazemi, F., & Hosseinpour, N. (2022). GIS-based land-use suitability analysis for urban agriculture development based on pollution distributions. Land Use Policy, 123, 106426. <https://doi.org/10.1016/j.landusepol.2022.106426>

18.	Andrea Zaniboni, Patrizia Tassinari, Daniele Torreggiani, GIS-based land suitability analysis for the optimal location of integrated multi-trophic aquaponic systems, Science of The Total Environment, Volume 913, 2024, https://doi.org/10.1016/j.scitotenv.2023.169790.

19.	Mohamed E.M. Jalhoum, Mostafa A. Abdellatif, Elsayed Said Mohamed, Dmitry E. Kucher, Mohamed Shokr, Multivariate analysis and GIS approaches for modeling and mapping soil quality and land suitability in arid zones, Heliyon, Volume 10, Issue 5, 2024, https://doi.org/10.1016/j.heliyon.2024.e27577.

35.	Sarasie Tennakoon, Armando Apan, Tek Maraseni, Richard Dein D. Altarez, Decoding the impacts of space and time on honey bees: GIS based fuzzy AHP and fuzzy overlay to assess land suitability for apiary sites in Queensland, Australia, Applied Geography, Volume 155, 2023, https://doi.org/10.1016/j.apgeog.2023.102951.

36.	Lian Xue, Peng Cao, Deze Xu, Ying Guo, Qingfang Wang, Xingfei Zheng, Ruijuan Han, Aiqing You, Agricultural land suitability analysis for an integrated rice–crayfish culture using a fuzzy AHP and GIS in central China, Ecological Indicators, Volume 148, 2023, https://doi.org/10.1016/j.ecolind.2022.109837.

**Comments:**

*3- Please recheck the quality of all figures in the paer becase they are not appeared in the file submitted.*

**Response:** We thank you for this suggestion. Please see inserted figures in main body text: figures in line 169 (Fig 1), in line 265 (Fig 2) and in line 392 (Fig 3)

**Action:** Three figures inserted in the article and as well attached on article submission system were upgraded. They have now more resolution and good quality.

**Response for Reviewer #2**

**We are very grateful to reviewer 2 for detailed and thoughtful comments which we find to be constructive and delivered professionally and appropriately. Thank you very much for your comments, each of which is addressed in the following. We have addressed all the comments that are now reflected in our revised manuscript and inserted line numbers and highlighted significant changes by using GREEN text highlight color to expedite the checking process.**

**Comments:**

*1) Explain* *how land suitability analysis contributes to understanding or resolving the problems related to particular post-socialist cities.*

**Response:** Thank you very much for the constructive comment. It has helped us to significantly improve the introduction section of the paper.

**Action: Added and modified in Line 68-103 as below:**

Furthermore, the typical monocentric spatial arrangement of the city was increasingly equipped in the city center throughout the Soviet era [9]. A significant population increase following the democratic revolution in 1990 created unregulated pressure on the city's centralized spatial structure. Additionally, Bertaud [10] examined the spatial organization of cities in Central and Eastern Europe (CEE) and discovered that these regions have a monocentric spatial structure. Following market transitions, it observed a gradual transition to polycentric structures. Despite limited research on the monocentric spatial structure of Ulaanbaatar city, there is a noticeable presence of this kind of transition in this area..

Urban planning is consistently linked to a variety of decision-making difficulties and strategies, and the results of planning will differ based on the combination of the approaches and techniques employed. During the Soviet era, Mongolia, like many other communist nations, employed planning approaches and techniques to design cities without involving citizens. This resulted in a simple, quick, and interruption-free planning procedure during the project [11].

In our knowledge, in the Soviet era, a monocentric structure allowed the communist government to “control everything” related to urban management. According to the centralized economy, it was cost-effective and easy to manage particularly to build mega infrastructure and new residential district. The urban development process was implemented under “bulldozer urbanism” approaches, whereas all land had been owned by the state. There was no need for residents' consent or negotiations with them to expropriate their lands. The municipality directly canceled the resident permit and gave notice to vacate the new development area by official declaration. This procedure referred to as “enter to ground” characterizes communist regimes' relation to their people. For the new development areas, only the land capability for construction was considered, focusing on investigating the physical characteristics of ground soil and topography.

These days, many of these nations have new laws requiring public engagement while also using new approaches and technologies. One of the most difficult tasks facing communities and municipal governments today is to locate prospective locations for the sustainable growth of city subcenters without threatening human rights and environmental protection. Land suitability analysis offers tools to address these management problems [11].

Ulaanbaatar city’s monocentric structure hinders sustainable development promoting traffic jams, air pollution, land grabbing, etc. Several international and local experts suggested to replan and switch from monocentric to polycentric structures by developing new city subcenters in Ulaanbaatar [7,11,12].

In short, there has been a constant increase in interest in adopting land suitability analysis since it can handle and combine many types of data (both spatial and non-spatial, as well as multi-temporal and multi-scale) in an efficient manner. Then, from a technical perspective, comprehension of the fundamental issue, and democratic points of view can all benefit the planning process [11].

**Comments:**

*2) Lines 158-163, the crime rate is classified into five classes based on the parameters used in Table 3. However, there is no explanation or explanation for the assumption you took in order to define a category as "high" or "very high" as in the case of crime rate "high" belongs to 0-0.6, why not 0-0.5? The selection of your parameters and how they are valued is an important aspect of your research. The sources for Table 3 are also lacking.*

**Response:** Thank you very much for the thoughtful comment. We followed your comment and added explanation on crime rate classification with used sources. The sources of Table 3 are added under it.

**Action: Added and modified in Line 213-215 as below:**

The crime maps data are categorized using a modified version of the Jenks natural breaks classification method, aiming to minimize within-group variance and maximize between-group variance [44]. Crime rate shows a percentage of recorded crime per population in each suburb and was categorized into five classes from low to high.

**In line 688 in the reference:**

44.	Chen, J., Yang, S. T., Li, H. W., Zhang, B., and Lv, J. R.: Research on Geographical Environment Unit Division Based on the Method of Natural Breaks (Jenks), Int. Arch. Photogramm. Remote Sens. Spatial Inf. Sci., XL-4/W3, 2013. 47–50, https://doi.org/10.5194/isprsarchives-XL-4-W3-47-2013,

**In line 256-257**:

All the classification methods of criteria are based on literature sources in Table 2 and below presented criteria classifications in detail (Table 3).

**In line 255:**

**Table 3. Parameters used in the analysis of residential suitability.**

| **Main criteria** | **Sub-criteria** | | | | | |
| --- | --- | --- | --- | --- | --- | --- |
| **Criteria classification** | | | | | | |
|  |  | 5 | 4 | 3 | 2 | 1 |
|  |  | High | Moderate | Medium | Low | Very Low |
| ***Livability*** | 1. Crime rate (%) | *0 - 0.6* | *0.7 - 1.2* | *1.3 - 2.0* | *2.1 - 3.2* | *3.3 - 4.4* |
|  | 2. Land use (code) | *11* | *61* | *24 & 23* | *21 - 23* | *13 - 16, 26 - 42, 63 - 65* |
| ***Accessibility*** | 3. Facility cover (%) | *0.18 - 4.18* | *4.19 - 9.42* | *9.43 - 15.60* | *15.61 - 23.07* | *23.08 - 38.87* |
|  | 4. Building cover (%) | *0 - 8.2* | *8.2 - 21.2* | *21.2 - 43.4* | *43.4 - 96.4* | *96.4 - 176.9* |
| ***Affordability*** | 5. Land price (₮ per square m) | < 48808 | 48809 -95549 | 95559 – 180393 | 180394 – 505308 | 505309 - 857143 |
| ***Fuzzy membership functions*** | | | | | | |
|  |  | Upper limit | Lower limit | Fuzzy functions | | |
|  | 6. Slope (degree) | *20* | *0* | *Decreasing linear* | | |
| ***Livability*** | 7. Distance from main park (m) | *10000* | *0* | *Decreasing linear* | | |
|  | 8. Distance from air pollution monitoring point (m) | *0* | *5000* | *Increasing linear* | | |
|  | 9. Distance from water protection zones (m) | *50* | *0* | *Boolean* | | |
| ***Accessibility*** | 10. Distance from school (m) | *3000* | *0* | *Decreasing linear* | | |
|  | 11. Distance from kindergarten (m) | *3000* | *0* | *Decreasing linear* | | |
|  | 12. Distance from hospital (m) | *5000* | *0* | *Decreasing linear* | | |
|  | 13. Distance from bus stop (m) | *1000* | *0* | *Decreasing linear* | | |
|  | 14. Distance from shopping center (m) | *2000* | *0* | *Decreasing linear* | | |
|  | 15. Distance from central and sub-central business district (m) | *5000* | *0* | *Decreasing linear* | | |
|  |  |  |  |  | | |

Data sources: Crime rate were generated from the open data of NSO’s website [43], land use, facility cover, building cover, land price, distance from main park, distance from water protection zones, distance from shopping centre were provided by ALMGC through the open-spatial data online portal [40], distance from air pollution point, distance from school, kindergarten, hospital, bus stop are processed data of the NUM-Lab.RaS [42], slope degree information was gathered from a subset of an optical stereo-based ASTER GDEM elevation data as well as radar-based SRTM elevation data [39,41], distance from CBD information based on JICA study [7].

**Comments:**

*3) Line 212. Could you please explain why the gamma coefficient is 0.9?*

**Response:** Thank you for your detailed and well-thought-out review. We followed the reviewers’ comment and added explanation on gamma coefficient number with based literature sources.

**Action: Added and modified in Line 278-280 as below:**

Zimmermann and Zysno [47] discussed a variety of fuzzy overlay operations and highlighted five useful operators to combine independent datasets with the fuzzy AND, fuzzy OR, fuzzy algebraic product, fuzzy algebraic sum and fuzzy γ -operators. Based on the studies of suitability analysis, the results showed that a 0.9-0.95 fuzzy gamma coefficient has a high accuracy for the suitability map [53, 54, 55].

In Line 713-722 at the reference list

53.	Kumar, R., Anbalagan, R. Landslide susceptibility zonation in part of Tehri reservoir region using frequency ratio, fuzzy logic and GIS. J Earth Syst Sci 124, 431–448 (2015). https://doi.org/10.1007/s12040-015-0536-2.

54.	Baharvand, S., Rahnamarad, J., Soori, S. et al. Landslide susceptibility zoning in a catchment of Zagros Mountains using fuzzy logic and GIS. Environ Earth Sci 79, 204 (2020). https://doi.org/10.1007/s12665-020-08957-w.

55.	Djouher Saadoud, Mohamed Hassani, Francisco José Martin Peinado, Mohamed Saïd Guettouche, Application of fuzzy logic approach for wind erosion hazard mapping in Laghouat region (Algeria) using remote sensing and GIS, Aeolian Research, Volume 32, 2018, Pages 24-34, https://doi.org/10.1016/j.aeolia.2018.01.002.

**Comments:**

*4) Line 411, what is the* *free land policy? Can you describe how it significantly affects the urban expansion and relate it to your results?*

**Response:** We thank you for this valuable comment and for pointing us to do the clear explanation on the result of our study. It has helped us to significantly improve the paper.

**Action: Added and modified in Line 495-513 as below:**

In term of the unsuitable residential area (99.93 km2), it’s expanding significantly [6,71] due to rural migration and free land policy implementation after 2002 [2,52]. The Law on Land (2002) grants Mongolian citizens, regardless of age and sex, one free land plot until May of 2028, in Ulaanbaatar city. According to the Land Inventory report [72], 277537 citizens owned land, and it was only 16.7 % of the residents of the capital city. Despite the abundance of land plots owned by residents, there is still a significant need for 9715.5 hectares of land in Ulaanbaatar city [74]. The impact of free land policy is severe in the city, even though it is a market economy, residential ger area land is administratively allocated free for residents without a proper urban plan and basic infrastructures [72,73,74]. Once the land was allocated, it was almost never recycled since the difficulties of legislation for land-use recycle and urban development on the government decision [49]. The free land policy resulted in the absence of land prices, removing all economic incentives to the land use recycling process in the city [49, 72].

As a result of this phenomenon, low-rise ger area continuously expands in the urban periphery with a low living condition, accelerating multidimensional poverty. Moreover, it is forming a spatial pattern of a concentric ring in the city and added by this process. Similarly, in Moscow and CEE cities, this phenomenon was observed and noted in the persistence and uniformity of housing types in successive rings [9,49]. Furthermore, whether in ger area or in sub-centre, households tend to be concentrated in the periphery with a low developed infrastructure, this spatial pattern is led to the increase of costs of living and pollutions.

**New citations added in Line 766-775 as Reference and as below**

72. The World Bank, Land Administration and Management in Ulaanbaatar, Mongolia, final report, 2018, Washington, USA, Available online: https://www.worldbank.org/en/country/mongolia/publication/land-administration-and-management-in-ulaanbaatar-mongolia.

73. The General authority for Land administration, geodesy and cartography of Mongolia, Land Inventory Report of Mongolia, 2024, Ulaanbaatar, Mongolia, Available online: https://egazar.gov.mn/api/landuse/nav.

74. Gombodorj G, 2023, 20 years of Land ownership of Mongolia (power point presentation), the conference of 20 years anniversary of Land ownership, Ulaanbaatar city Government Palace, Mongolia.

**Comments:**

*5) On line 426 of your conclusion, you mentioned that the* *city spatial pattern of residential suitability is changing, however you did not describe how it has changed over time.*

**Response:** Thank you for bringing up the interesting idea to describe residential suitability change in details. In response to your comment, we now highlighted the change of the residential suitability in discussion section and changed way of concluding words in the conclusion.

**Action: Added and modified in Line 452-480 and 533 as below:**

Ulaanbaatar city has demonstrated a similar spatial pattern of residential suitability. Since the economic capital of the citizens has increased, mid and high-societal groups are starting to prefer urban fringes or preurban agricultural areas for residential propose, compared to the socialist era, where CBDs were in high demand. Ulaanbaatar City's housing market immediately reflected that kind of "close to nature" choice and private land developers started small residential development projects in suitable urban fringes, which are gradually changing an urban monocentric structure to a polycentric one.

It’s evident, that Ulaanbaatar is the oldest urban center in Mongolia with a history of more than 700 years. The monocentric structure of the city had been created and administrated without land market implications during the Soviet era, with most urban growth of that time. This period saw land nationalization and centralized administrative allocation, impacting the internal spatial pattern of the city defined as prominent centers with extensive, radial, and concentric transit networks reinforcing their monocentric structure [72]. The combination of the centralized location of the national institutions, the main role in the domestic economy, and the high rate of urbanization in the post-socialist city founded insecure property rights, institutional disorganization, governance problems, and weak urban planning regulations. Mongolia’s transition to a market economy was formed in the 1990s through the legal reform of numerous legal frameworks about land and property, including the Land Law, the Law on Allocation of Land to Citizens of Mongolia for Ownership, and the Law of Urban Development. Initially, the government proposed free land policy accelerated the market economy, helped the reduction of poverty, and allowed poor urban residents of slum districts (ger area) to own valuable residential land plots as immovable property. However, the free land policy harms urban management, accelerates urban expansion in city fringes, and creates difficulties in infrastructure construction, private owners refuse new development plans, increasing land speculation and corruption in the public sector.

The legal frameworks empower the local government of the capital city to organize land allocation, and public participatory planning principles are to be applied and reflected in urban development plans and activities [72,73,74]. Even though Mongolia has changed its urban regulation and administrative system, the historically centralized administration on the monocentric structure somehow exists and impacts the urban expansion on spatial patterns [9].

**In line 533 as below:**

To conclude, key changes are observed from the results, and it can be said that the city spatial pattern of residential suitability is changing to polycentric structure slowly.

**Comments:**

*6) As this is a land suitability analysis study, one would expect to see more of a focus on how post- or pre-socialist urban policies contribute to land expansion and suitability. In this manner, policymakers will be able to use the findings to formulate strategies that can be implemented.*

**Response:** We thank you for this suggestion, which led to the detailed text additions in discussion section.

**Action: Added and modified in Line 419-480 and as below:**

However, it can be said that those matching boundaries could be representing a weak feature of centralized infrastructure development, including road network, heating supply network, water supply network, sewerage network and the railroad network, and accessibility of basic services. Historically, the ’city’s spatial structure is traditional monocentric, which tend to more equipped in the city centre in the Soviet era [9,10,49]. After the democratic revolution in 1990, a lack of urban development policies and immature land market during the unprecedented period with significant population growth pressured to form the uncontrolled and centralized formation of spatial structure in the city. However, it is unclear to define the border of the developing area of the city, caused of imprecise components and a subjective methodology. As a result, it enables a subjective influence on urban plans. Moreover, urban planners and decision-makers have acknowledged a circumstance that city problems related to environment, society, economy, transportation, infrastructure, and spatial planning are not possible to fully determine before the planning process. One of the prime steps in formulating the Ulaanbaatar master plan is “an evaluation of complex assessment for urban development”. However, in this assessment, 65 factors of 4 categories, including environmental (26), societal and economic (20), infrastructural (8), architectural, and landscape (8) are evaluated, and the scoring is limited from 1 to 5 with arithmetic average. Furthermore, the scoring of the evaluation did not recognize the spatial parameters (geometry). As a result, assessment is not possible due to inadequate information for spatial planning. Therefore, the proposed methodology should be made essential in planning projects, since our study's results clearly show the benefits of taking residential suitability analysis using fuzzy logic into consideration when examining suitability for new residential area development.

Furthermore, Bertaud [10] analyzed the spatial structure of Central and Eastern European (CEE) cities and found that it has a monocentric spatial structure causing a centralized command economy, maintaining their prestigious cultural centre, and converting to a polycentric structure slowly after their market transitions. Generally, one of the key changes in our results is that the monocentric spatial pattern is converting slowly into a polycentric structure (Fig 3). Regarding a high-rise apartment residential area, surprisingly, CBD is not identified as the most and second suitable site according to our findings, and the pattern of CBD has been changing to be mixed and converting to multi-purposes activities [3,6]. Moreover, the value of the residential area in CBD is slowly dropping due to inappropriate living condition, including a high density of building, underdeveloped public transportation, lack of green space and air pollution, indicating to develop and maintain in CBD as a cultural or business centre. UN [1] also noted this urbanization consequences, and government policy, regulation on urban development and investment responses market liabilities are the primary victims based on the CEE cities [1, 9]. Moreover, the real estate market plays a vital role in city development, open information about prices, the business environment of real estate agent, clear land and property tenure, property registration institution, the flexibility of land use change, subsidies and taxation policy [49]. Ulaanbaatar city has demonstrated a similar spatial pattern of residential suitability. Since the economic capital of the citizens has increased, mid and high-societal groups are starting to prefer urban fringes or preurban agricultural areas for residential propose, compared to the socialist era, where CBDs were in high demand. Ulaanbaatar City's housing market immediately reflected that kind of "close to nature" choice and private land developers started small residential development projects in suitable urban fringes, which are gradually changing an urban monocentric structure to a polycentric one.
